# Supplementary material for: Estrogen receptor positive breast cancers have patient specific hormone sensitivities and rely on progesterone receptor
Source: Nat Commun. 2022 Jun 6;13:3127. doi: 10.1038/s41467-022-30898-0 (PMC9170711; doi:10.1038/s41467-022-30898-0)
Supplement: Supplementary file 1 — Supplementary Information [file 41467_2022_30898_MOESM1_ESM.pdf]

Supplementary Table 1: Plasmid description

Plasmid	Backbone	Sequence/Reference
sg*PGR_1*	CRISPR-Cas9_lenti_v2	CACCGTTCTTTAAGAGGGCAATGGA
sg*PGR_2*	CRISPR-Cas9_lenti_v2	CACCGCAGCACAACTACTTATGTGC
sh*ESR1*	pLKO.1	TRCN0000003300
shPGR	pLKO.1	TRCN0000003324
hPGR	pIX304	cloned from the ORFeome collection

Supplementary Table 2: Hormone pellet composition

Hormone pellet	Cat. #	Hormone dosage per pellet (mg)	Silicon Part A mg	Silicon Part B ul	Hormone powder (mg)	Pellet (mg) per animal
E2	E2758	0.3	4700	500	250	7.8
P4	P0130-25G	20	3525	375	3525	49.5
