# Supplementary material for: Estrogen receptor positive breast cancers have patient specific hormone sensitivities and rely on progesterone receptor
Source: Nat Commun. 2022 Jun 6;13:3127. doi: 10.1038/s41467-022-30898-0 (PMC9170711; doi:10.1038/s41467-022-30898-0)
Supplement: Supplementary file 3 — Reporting Summary [file 41467_2022_30898_MOESM3_ESM.pdf]

nature portfolio

Corresponding author(s): Prof. Cathrin Brisken
Last updated by author(s): Apr 17, 2022

Reporting Summary

Nature Portfolio wishes to improve the reproducibility of the work that we publish. This form provides structure for consistency and transparency in reporting. For further information on Nature Portfolio policies, see our Editorial Policies and the Editorial Policy Checklist.

Statistics

For all statistical analyses, confirm that the following items are present in the figure legend, table legend, main text, or Methods section.

n/a	Confirmed	
☐	☒	The exact sample size (*n*) for each experimental group/condition, given as a discrete number and unit of measurement
☐	☒	A statement on whether measurements were taken from distinct samples or whether the same sample was measured repeatedly
☐	☒	The statistical test(s) used AND whether they are one- or two-sided<br>*Only common tests should be described solely by name; describe more complex techniques in the Methods section.*
☐	☒	A description of all covariates tested
☐	☒	A description of any assumptions or corrections, such as tests of normality and adjustment for multiple comparisons
☐	☒	A full description of the statistical parameters including central tendency (e.g. means) or other basic estimates (e.g. regression coefficient) AND variation (e.g. standard deviation) or associated estimates of uncertainty (e.g. confidence intervals)
☐	☒	For null hypothesis testing, the test statistic (e.g. *F*, *t*, *r*) with confidence intervals, effect sizes, degrees of freedom and *P* value noted<br>*Give P values as exact values whenever suitable.*
☒	☐	For Bayesian analysis, information on the choice of priors and Markov chain Monte Carlo settings
☒	☐	For hierarchical and complex designs, identification of the appropriate level for tests and full reporting of outcomes
☒	☐	Estimates of effect sizes (e.g. Cohen's *d*, Pearson's *r*), indicating how they were calculated

Our web collection on statistics for biologists contains articles on many of the points above.

Software and code

Policy information about availability of computer code

Data collection	Live imaging was performed with Xenogen IVIS Imaging System 200 (Caliper Life Sciences).<br>Ventana Discovery ULTRA (Roche Diagnostics, Rotkreuz, Switzerland) with Ventana solutions for IHC staining.<br>Histological slides were scanned with Olympus VS120-L100 slide scanner using a 20x/0.75 objective connected to a Pike F505 C Color camera.<br>Hormone measurements were assessed using liquid chromatography-mass spectrometry as described in 51.<br>Stereo micrographs were imaged by LEICA MZ FLIII stereomicroscope with Leica MC170 HD camera, fluorescence images were acquired with LEICA M205FA fluorescence stereomicroscope equipped with a Leica DFC 340FX camera.<br>RNAseq was performed using Illumina HiSeq with 2x150bp configuration.
Data analysis	Living Image Software (v4.7.3), https://www.perkinelmer.com/uk/lab-products-and-services/resources/in-vivo-imaging-software-downloads.html<br>QuPath (v0.1.4) , https://qupath.github.io/) using the BioFormats Extension (https://github.com/qupath/qupath/bioformats-extension).<br>Fiji/ImageJ (v1.52t), https://imagej.nih.gov/ij/<br>HISAT2 (v2.1.0), http://daehwankimlab.github.io/hisat2/<br>FeatureCounts (v2.0.0), http://subread.sourceforge.net/)<br>Bioconductor v3.8, https://bioconductor.org/<br>edgeR (v3.24.0), http://bioconductor.org/packages/release/bioc/html/edgeR.html<br>Limma (v3.38.0), https://bioconductor.org/packages/release/bioc/html/limma.html<br>ClusterProfiler (v4.0.0), https://bioconductor.org/packages/release/bioc/html/clusterProfiler.html<br>MSigDB (v6.2), https://www.gsea-msigdb.org/gsea/msigdb/<br>GraphPad Prism 8.0, https://www.graphpad.com/scientific-software/prism/<br>R (v3.5.3), https://www.r-project.org/<br>Nlme package (v3.1-143), https://cran.r-project.org/web/packages/nlme/index.html<br>LI-COR Image Studio™ Lite Software, https://www.licor.com/

Thermo Xcalibur (v4.0.27.10), https://www.thermofisher.com/order/catalog/product/OPTON-30965

For manuscripts utilizing custom algorithms or software that are central to the research but not yet described in published literature, software must be made available to editors and reviewers. We strongly encourage code deposition in a community repository (e.g. GitHub). See the Nature Portfolio guidelines for submitting code & software for further information.

Data

Policy information about availability of data

All manuscripts must include a data availability statement. This statement should provide the following information, where applicable:

- Accession codes, unique identifiers, or web links for publicly available datasets
- A description of any restrictions on data availability
- For clinical datasets or third party data, please ensure that the statement adheres to our policy

The authors declare that all data supporting this study's findings are available within this manuscript and its supplementary files.
RNAseq datasets used in this study have been deposited in GEO and can be accessed via the following accession numbers: GSE192808, GSE192809, GSE192810

Field-specific reporting

Please select the one below that is the best fit for your research. If you are not sure, read the appropriate sections before making your selection.

☒ Life sciences ☐ Behavioural & social sciences ☐ Ecological, evolutionary & environmental sciences

For a reference copy of the document with all sections, see nature.com/documents/nr-reporting-summary-flat.pdf

Life sciences study design

All studies must disclose on these points even when the disclosure is negative.

Sample size	Sample size was mainly determined based on resource constraints. Non a priori power analysis has been performed. Nevertheless, for choosing the sample size in our study we took into account the effect size and variability of our experimental conditions.
Data exclusions	All replicates were included in this study and they were all successful. Hence, no exclusion of data was performed.
Replication	All in vitro and in vivo experiments were performed in replicates. Most experiments were repeated at least three times.
Randomization	The experimental animals were randomized for their allocation in groups based on their baseline radiance signal.
Blinding	Blinding was not possible for in vivo experiments as a uniform baseline radiance signal was required for the randomization of the animals in experimental groups.

Reporting for specific materials, systems and methods

We require information from authors about some types of materials, experimental systems and methods used in many studies. Here, indicate whether each material, system or method listed is relevant to your study. If you are not sure if a list item applies to your research, read the appropriate section before selecting a response.

Materials & experimental systems

n/a	Involved in the study	
☐	☒	Antibodies
☐	☒	Eukaryotic cell lines
☒	☐	Palaeontology and archaeology
☐	☒	Animals and other organisms
☐	☒	Human research participants
☒	☐	Clinical data
☒	☐	Dual use research of concern

Methods

n/a	Involved in the study	
☒	☐	ChIP-seq
☒	☐	Flow cytometry
☒	☐	MRI-based neuroimaging

Antibodies

Antibodies used

Primary antibodies for western blot
ER for WB, Santa Cruz, cat.# sc-543. Dilution: 1:1000
PR for WB, Santa Cruz, cat.# sc-7208. Dilution: 1:1000
Cyclin D1, Neomarkers, cat.# RB-212-P0. Dilution: 1:1000

Secondary antibodies for IHC

Rabbit Impress HRP antibody, Vector laboratories, cat.# MP-7401. Dilution: 1:700.

Primary antibodies for IHC
ER for IHC, Zytomed System, cat.# BRB053. Ready-to-use solution.
PR for IHC, Ventana, cat.# 790-2223. Ready-to-use solution.
Ki67 Abcam, cat.# M3060. Dilution: 1:400.
pHH3, Abcam, cat.# ab5176. Dilution: 1:5000.

Secondary antibodies for western blot
goat anti-rabbit immunoglobulins/HRP (cat# P0448, DAKO). Dilution: 1:4000.
goat anti-mouse immunoglobulins/HRP (cat# P0447, DAKO). Dilution: 1:4000.

Loading control for western blot
γ-tubulin, Sigma, clone GTU-88, cat.# T5326. Dilution 1:10000.

Validation

Antibodies were validated by the manufacturers:
Zytomed Service: https://www.zytomed-systems.com/ , ER for IHC: https://www.zytomed-systems.com/storage/uploads/datasheets/en/BRB053_EN_Gef.pdf
Santa Cruz: https://www.scbt.com/home , ER for WB: https://www.scbt.com/it/p/eralpha-antibody-hc-20 , PR for WB: https://www.scbt.com/it/p/pr-antibody-h-190
Roche: https://www.roche.com/ , PR for IHC: https://www.labome.com/product/Ventana/790-2223.html
Abcam: https://www.abcam.com/ , Ki67: https://lifescience.roche.com/documents/M306_RUO_E.pdf , pHH3: https://www.abcam.com/histone-h3-phospho-s10-antibody-ab5176.html
Neomarkers: https://www.thermofisher.com/ch/en/home.html , Cyclin D1: https://assets.thermofisher.com/TFS-Assets/APD/Specification-Sheets/D11682~.pdf
Vector Laboratories: https://vectorlabs.com/, anti-rabbit Impress HRP: https://vectorlabs.com/products/enzyme-polymer/immpress-hrp-horse-anti-rabbit-igg
Agilent Dako: https://www.agilent.com/en/dako-products , goat anti-rabbit: https://www.agilent.com/en/product/immunohistochemistry/antibodies-controls/secondary-antibodies/goat-anti-rabbit-immunoglobulins-hrp-(affinity-isolated)-153244 , goat anti-mouse: https://www.agilent.com/en/product/immunohistochemistry/antibodies-controls/secondary-antibodies/goat-anti-mouse-immunoglobulins-hrp-(affinity-isolated)-153239
Sigma-Aldrich: https://www.sigmaaldrich.com/ , γ-tubulin: https://www.sigmaaldrich.com/CH/de/product/sigma/t5326?gclid=CjwKCAjwur-SBhB6EiwA5sKtjiZveT1dT3JoaBOnjy7uts13Y1Vmfzec0Pp-jKQBFR7XoOyQ0YZ03RoCZVUQAvD_BwE

Eukaryotic cell lines

Policy information about cell lines

Cell line source(s)

ATCC Cell Lines: MCF7, T47D, HCC1428, MDA-MB-134-VI.

Authentication

Cell lines have been authenticated by ATCC and confirmed by morphological analysis using light microscopy.

Mycoplasma contamination

Cells are regularly checked for mycoplasma contamination and there was no mycoplasma contamination for the cell lines used in this study.

Commonly misidentified lines
(See ICLAC register)

Commonly misidentified cell lines were not used in the study.

Animals and other organisms

Policy information about studies involving animals; ARRIVE guidelines recommended for reporting animal research

Laboratory animals

NOD.Cg-Prkdcscid Il2rgtm1Wjl482 /SzJ (NSG) mice were purchased from Charles River. Female mice were mantained and handled according to Swiss guidelines. Female mice used for this study were intraductally xenografted when they were 8 to 12 weeks old.

Wild animals

Wild animals were not used in this study.

Field-collected samples

Field-collected samples were not used in this study.

Ethics oversight

All animal experiments were performed in accordance with protocols approved by the Service de la Consommation et des Affaires Vétérinaires of Canton de Vaud, Switzerland (VD 1865.3, 1865.4, 1865.5).

Note that full information on the approval of the study protocol must also be provided in the manuscript.

Human research participants

Policy information about studies involving human research participants

Population characteristics

Patients whose tumors were used to establish xenografts in this study have the following characteristics:
T84: female, 57 years old, ER+PR+HER2- tumor, naive
T99: female, 57 years old, ER+PR+HER2- tumor, naive
T105: female, 59 years old, ER+PR+HER2- tumor, naive
T109: female, 44 years old, ER+PR+HER2- tumor, naive
T110: female, 49 years old, ER+PR+HER2- tumor, naive

T111: female, 44 years old, ER+PR+HER2- tumor, naive
T113: female, 70 years old, naive
PL-011: female, 50, ER+PR+HER2- tumor, advanced stage
PL-015: female, 59, ER+PR+HER2- tumor, advanced stage
Further information about patient-derived tumors used in this study are detailed in Supplementary Table 1.

Recruitment	No bias was applied in the recruitment process of patients whose biological material was used for this study.<br>Inclusion criteria for primary tumor: Invasive carcinoma, larger than 1.5 centimeter, without treatment or refractory to treatment, negative viral tests for HIV and Hepatitis B & C.<br>Exclusion Criteria: Carcinoma < 1.5 centimeter, non-invasive, viral tests positive.
Ethics oversight	All subjects participating in the clinical investigation provided written informed consent and the study protocol was approved by the Commission cantonale d'éthique de la recherche sur l'être humain (45-05 and 72-04).

Note that full information on the approval of the study protocol must also be provided in the manuscript.
